# Targeted metabolomics identifies accurate CSF metabolite biomarkers for the differentiation between COVID-19 with neurological involvement and CNS infections with neurotropic viral pathogens

**DOI:** 10.1186/s12967-024-05422-1

**Published:** 2024-07-03

**Authors:** Frieder Neu, Sandra Nay, Sven Schuchardt, Frank Klawonn, Thomas Skripuletz, Kurt-Wolfram Suehs, Frank Pessler

**Affiliations:** 1 Research Group Biomarkers for Infectious Diseases, TWINCORE Centre for Experimental and Clinical Infection Research, Feodor-Lynen-Str. 7, 30625 Hannover, Germany; 2 Study Programme Medicine, Riga Stradins University, Riga, Latvia; 3 Department of Neurology, Hannover Medical School, Hannover, Germany; 4 Fraunhofer Institute for Toxicology and Experimental Medicine (ITEM), Hannover, Germany; 5 Biostatistics, Helmholtz Centre for Infection Research, Brunswick, Germany; 6 Centre for Individualised Infection Medicine, Hannover, Germany; 7 Research Group Biomarkers for Infectious Diseases, Helmholtz Centre for Infection Research, Brunswick, Germany

**Keywords:** Biomarker, Ceramides, Cerebrospinal fluid, COVID-19, Diagnosis, Encephalitis, Long COVID, Meningitis, Metabolism, Long-COVID, Neuroinflammation, SARS-CoV-2, Triglycerides

## Abstract

**Background:**

COVID-19 is primarily considered a respiratory tract infection, but it can also affect the central nervous system (CNS), which can result in long-term sequelae. In contrast to CNS infections by classic neurotropic viruses, SARS-CoV-2 is usually not detected in cerebrospinal fluid (CSF) from patients with COVID-19 with neurological involvement (neuro-COVID), suggesting fundamental differences in pathogenesis.

**Methods:**

To assess differences in CNS metabolism in neuro-COVID compared to CNS infections with classic neurotropic viruses, we applied a targeted metabolomic analysis of 630 metabolites to CSF from patients with (i) COVID-19 with neurological involvement [n = 16, comprising acute (n = 13) and post-COVID-19 (n = 3)], (ii) viral meningitis, encephalitis, or myelitis (n = 10) due to herpes simplex virus (n = 2), varicella zoster virus (n = 6), enterovirus (n = 1) and tick-borne encephalitis virus (n = 1), and (iii) aseptic neuroinflammation (meningitis, encephalitis, or myelitis) of unknown etiology (n = 21) as additional disease controls.

**Results:**

Standard CSF parameters indicated absent or low neuroinflammation in neuro-COVID. Indeed, CSF cell count was low in neuro-COVID (median 1 cell/µL, range 0–12) and discriminated it accurately from viral CNS infections (AUC = 0.99) and aseptic neuroinflammation (AUC = 0.98). 32 CSF metabolites passed quality assessment and were included in the analysis. Concentrations of differentially abundant (fold change ≥|1.5|, FDR ≤ 0.05) metabolites were both higher (9 and 5 metabolites) and lower (2 metabolites) in neuro-COVID than in the other two groups. Concentrations of citrulline, ceramide (d18:1/18:0), and methionine were most significantly elevated in neuro-COVID. Remarkably, triglyceride TG(20:1_32:3) was much lower (mean fold change = 0.09 and 0.11) in neuro-COVID than in all viral CNS infections and most aseptic neuroinflammation samples, identifying it as highly accurate biomarker with AUC = 1 and 0.93, respectively. Across all samples, TG(20:1_32:3) concentration correlated only moderately with CSF cell count (ρ = 0.65), protein concentration (ρ = 0.64), and Q-albumin (ρ = 0.48), suggesting that its low levels in neuro-COVID CSF are only partially explained by less pronounced neuroinflammation.

**Conclusions:**

The results suggest that CNS metabolite responses in neuro-COVID differ fundamentally from viral CNS infections and aseptic neuroinflammation and may be used to discover accurate diagnostic biomarkers in CSF and to gain insights into differences in pathophysiology between neuro-COVID, viral CNS infections and aseptic neuroinflammation.

**Supplementary Information:**

The online version contains supplementary material available at 10.1186/s12967-024-05422-1.

## Background

The coronavirus disease 19 (COVID-19) pandemic has resulted in significant morbidity and mortality worldwide with long-term sequelae [1–3]. Although primarily considered a respiratory disease, mounting evidence suggests that severe acute respiratory syndrome coronavirus 2 (SARS-CoV-2) infection can also affect the central nervous system (CNS). The symptoms reported range from gustatory and olfactory dysfunction over altered mental status to seizures and meningitis, encephalitis, and cerebrovascular events [4–7]. A recent multicenter study analyzed standard cerebrospinal fluid (CSF) parameters of 127 COVID-19 patients and found evidence of blood-CSF-barrier (BCB) dysfunction, a relative absence of neuroinflammation, and oligoclonal bands (intrathecal IgG) in a subset of patients. This study also reported that patients with severe COVID-19 and neurological symptoms had higher levels of protein and leukocytes in CSF than patients with mild symptoms [8]. Although the sensitivity of SARS-CoV-2 detection by naso-pharyngeal swab and reverse transcription-polymerase chain reaction (RT-PCR) is 97.2% and remains the gold standard in the diagnosis of COVID-19 [4], the detection of SARS-CoV-2 in the CSF through RT-PCR is quite inconsistent. Most studies have failed to confirm the presence of the virus in CSF [8–10]. However, in a small study with 11 participants, next-generation sequencing provided direct evidence for the presence of SARS-CoV-2 in the CSF [11], suggesting that the viral load in CSF may be below the sensitivity of conventional RT-PCR. This observation suggests that neuropathology in COVID-19 is not primarily driven by viral replication in CNS but by other, less direct processes resulting in neuroinflammation and neuronal dysfunction.

Thus, there is a need to identify host-dependent markers for COVID-19 CNS disease activity, both to improve diagnosis and to gain a deeper understanding of the pathophysiological mechanisms in the CNS. Indeed, analysis of changes in the CSF proteome of patients with COVID-19 detected increased levels of C-reactive protein (CRP), upregulation of the complement system and coagulation cascade, and perturbed neuronal growth and signaling, cell adhesion, and macrophage activation. The simultaneous involvement of these factors may contribute to the pathogenesis of neurocognitive dysfunction and neuroinflammation in SARS-CoV-2 infection [11].

Metabolomics has emerged as a promising tool to study pathophysiological changes associated with host–pathogen interaction, and to discover biomarkers for type and severity of CNS infection and to discriminate between transmissible and non-transmissible neuroinflammatory disorders [12–19]. Among the latter studies, Ratuszny et al. found clear changes in CSF metabolites in patients with enterovirus meningitis even when CSF cell count was normal [13]. Considering that CSF cell count in neuro-COVID is usually normal or only mildly elevated, investigating changes in CSF metabolites may constitute an additional approach to identifying disease-associated biomarkers. Indeed, a nontargeted metabolomic screen of the CSF of 10 patients with COVID-19 with acute-onset delirium revealed differentially abundant compounds related to protein catabolism, foodborne molecules, alcoholic beverages, micropollutants, and miscellaneous compounds. These findings suggest that lifestyle factors could represent risk factors for CNS dysfunction in individuals affected by COVID-19 [20]. Results from COVID-19 samples in that study were compared against 7 control subjects with “septic conditions”, which did not allow to search for differences between neuro-COVID and CNS infections by common neurotropic viral pathogens such as herpes simplex or varicella zoster viruses.

In the present study, we therefore applied a comprehensive targeted metabolomics/lipidomic analysis of CSF from patients with COVID-19 with neurological involvement and (i) patients with CNS infection due to a defined viral pathogen and (ii) patients with clinical evidence of encephalitis/meningitis/myelitis in whom a causative pathogen could not be detected.

## Methods

### Study cohort

The study was approved by the Ethics Committee of Hannover Medical School (MHH; file no. 3142-2016). CSF samples were collected between 2018 and 2021 in the Dept. of Neurology of MHH. The neuro-COVID samples were collected between March 2020 and June 2021. None of the participants had received a SARS-CoV-2 vaccine. The alpha variant was the predominant variant in Germany during the study period. One patient became infected after March 2021, when the delta variant was already predominant. The study cohort comprises patients diagnosed with (i) COVID-19 with neurological involvement (COVID-19, n = 16), of whom n = 13 were recruited in the acute stage and n = 3 in the post-COVID-19 stage, (ii) meningitis, encephalitis, or myelitis with detection of a viral pathogen (disease controls with viral pathogen, dCtrl [viral], n = 10; herpes simplex virus (HSV) encephalitis (n = 2), varicella zoster virus (VZV) meningoencephalitis (n = 5), VZV radiculomyelitis (n = 1), enterovirus meningitis (n = 1), and tick-born encephalitis (n = 1), and (iii) aseptic meningitis, encephalitis, or myelitis without viral pathogen detection or other defined etiology [disease controls with unknown etiology, dCtrl (unknown), n = 21], using clinical diagnostic criteria according to [21–23]. One possible COVID-19 sample was excluded because of a possible coinfection with another viral pathogen. Table S1 summarizes diagnostic criteria and case definitions. CSF was obtained by lumbar puncture, centrifuged for 15 min at 900 rounds per min to sediment cells and debris, using a Thermo Scientific™ Megafuge™ ST Plus Series with a TX-400 rotor (radius, 168 mm). Aliquots of supernatants were frozen at −80 °C within 2 h.

### Standard blood and CSF parameters

Peripheral blood leukocyte counts and CRP levels were measured in blood samples obtained at the time of lumbar puncture. The sample collection protocol is detailed in Suehs et al. [14]. The following standard CSF parameters were assessed: cell count, lactate concentration, protein concentration, Q-albumin (the ratio of CSF albumin to serum albumin), and IgG index (the ratio of IgG to Q-albumin). BCB disruption was evaluated using age-adjusted Q-albumin. No dysfunction: Q-albumin ≤ reference limit, calculated as (age/15) + 4; mild ≤ 15, moderate ≤ 25, severe > 25 [24].

### Targeted metabolomic profiling

Metabolite concentrations were measured on a SCIEX 5500 QTrapTM mass spectrometer (SCIEX, Darmstadt, Germany) using the MxP Quant 500 kit (Biocrates, Life Sciences AG, Innsbruck, Austria). The kit combines flow injection analysis tandem mass spectrometry (FIA-MS/MS) for lipids and liquid chromatography tandem mass spectrometry (LC–MS/MS) using Agilent 1290 Infinity II liquid chromatography (Santa Clara, CA, USA) coupled with a tandem mass spectrometer for small molecules. Depending on the sample to be analyzed, this kit allows the quantification of up to 630 metabolites: alkaloids (1), amine oxides (1), amino acids (20), amino acid related molecules (30), bile acids (14), biogenic amines (9), carboxylic acids (7), cresols (1), fatty acids (12), hormones and related (4), indoles and derivatives (4), nucleobases and related (2), vitamins and cofactors (1), carbohydrates and related (1), acylcarnitines (40), lysophosphatidylcholines (14), phosphatidylcholines (76), sphingomyelines (15), ceramides (28), dihydroceramides (8), hexosylceramides (19), dihexosylceramides (9), trihexosylceramides (6), cholesterol esters (22), diglycerides (44), and triacylglycerols (triglycerides) (242). Metabolite extraction and all analytical assays were conducted in accordance with the protocols provided by the manufacturer (https://biocrates.com/mxp-quant-500-kit, accessed on 05 June 2023). The MetIDQ™ software tool (Biocrates Life Science AG, Innsbruck, Austria) was used for peak integration and to calculate metabolite concentrations.

### Quality screen

The samples were measured on two 96 well plates. We performed a strict quality screen to maintain high quality of data and reduce sources of bias. To account for between-plate variability, we excluded all metabolites (i) whose concentrations on the two plates differed by more than 1.5-fold, with a false discovery rate (FDR) < 0.5, (ii) whose limit of detection (LOD, defined for most analytes as three times the signal obtained with the blank) differed between the two plates more than twofold, and (iii) which were > LOD exclusively on one plate. To further reduce biostatistical bias inherent to multiple hypothesis testing, we reduced the number of analytes (hypotheses) by including only those that were detected > LOD in the majority of samples of at least one group, as was done in our previous studies [12, 13]. Specifically, we included only analytes that were detected > LOD in at least 80% of either the neuro-COVID, dCtrl (viral) or dCtrl (unknown) samples. After this screening process, we included 32 analytes comprised of 3 acylcarnitines, 1 amine oxide, 12 amino acids, 5 amino acid related molecules, 1 biogenic amine, 3 carboxylic acids, 1 ceramide, 2 hexosylceramides, 1 dihexosylceramide, 2 nucleobase-related metabolites, and 1 triglyceride (Table S2). Any concentrations that were measured as < LOD were replaced with the pseudovalue LOD/2.

### Statistical analyses

We imputed missing values to ensure compatibility with statistical analyses that do not support missing values. 3 missing values for CSF lactate, and 2 for CSF protein were replaced by multiple imputation using SPSS Statistics for Windows, version 29 (SPSS, IBM Corporation,

Armonk, NY, USA; SPSS Missing Values Manual [25]) using diagnosis, age, sex, peripheral blood CRP and leukocyte count and CSF protein concentration, cell count, lactate concentration, Q-albumin, and IgG index as predictors to impute missing values. The resulting datasets were pooled to create a “pooled estimate” dataset. Values for standard parameters that were < LOD were replaced by LOD/2: CRP LOD = 0.6 mg/L, replaced with 0.3 mg/L; IgG LOD = 0.00926 g/L, replaced with 0.0045 g/L.

Nonparametric statistical tests were employed because of the non-normal distribution of the data. The Kruskal–Wallis test was used to compare median values across groups, while the Chi-squared (χ^2^) test and Fisher's exact test were used to evaluate differences in categorical variables using SPSS v29. Significance was considered at a *p* of < 0.05.

For principal component analysis (PCA), data were log_10_ transformed, but were not scaled. Differential abundance analysis was performed using the Mann–Whitney U test to compare median values between groups. The Benjamini–Hochberg correction was implemented with an FDR of 0.05 to account for multiple testing. A fold change (FC) threshold of ≥|1.5|, was set for differential abundance analysis. Receiver operating characteristic (ROC) curve analysis was employed to measure the discriminatory accuracy of biomarkers. Accurate biomarker candidates were defined as having an area under the ROC curve (AUC) ≥ 0.8, lower bound 95% confidence interval (CI) not crossing the chance line of 0.5, and an asymptotic *p* < 0.05. The Youden index method was used to calculate sensitivity, specificity, positive predictive value (PPV), and negative predictive value (NPV) at the optimal cut-off value. Spearman's rank correlation coefficient was used to assess correlations. PCA, differential abundance analysis, ROC curve analysis including the Youden index analyses, and correlation testing were performed using the open-source software MetaboAnalyst 5.0 (https://www.metaboanalyst.ca [26]). Internal cross-validation of biomarker candidates was performed using the leave-one-out (jackknife) procedure [27], using the frequency with which a given biomarker was selected among the top 5 or top 10 classifiers as the measure of validity.

## Results

### Demographic features and standard blood and CSF parameters

Table 1 summarizes sociodemographic and routine laboratory parameters. The dCtrl (viral) patients possessed the characteristics of viral CNS infections previously documented by us using a different patient cohort [8, 12, 13, 15]. The COVID-19 patients manifested less neuroinflammation than both disease control groups, as evidenced by lower CSF cell count, protein level, and IgG index, and a tendency towards less BCB disruption. On the other hand, the COVID-19 samples exhibited higher median blood CRP values than the disease controls, indicating more pronounced inflammation in the periphery but not in the CNS. Age and sex did not differ significantly among the three groups.

**Table 1:** Demographic data and standard blood and CSF parameters

	COVID-19	Disease Ctrl (viral)	Disease Ctrl (unknown)	*p* value
	Median (range)			
Demographic				
Age [years]	57 (31–81)	49.5 (26–79)	56 (19–79)	0.63^a^
Sex [female %]	31.3%	60.0%	47.6%	0.36^b^
Blood				
Leukocyte count [1000/µL]	6.0 (3.3–13.4)	6.6 (3.5–10.6)	7.8 (5.4–13.2)	0.12^a^
C-reactive protein [mg/L]	29.0 (0.3–216.9)	3.7 (0.3–74.5)	7.9 (0.3–284.4)	0.07^a^
CSF				
Lactate [mmol/L]	2.0 (1.3–3.9)	2.0 (1.6–3.8)	2.1 (0.2–4.0)	0.92^a^
Cell count [1/µL]	1.0 (0.3–12)	77.4 (12.0–549)	15.0 (4.0–397)	2.5E−07^a^
Protein [g/L]	0.4 (0.2–0.6)	0.5 (0.4–1.8)	0.6 (0.1–8.7)	1.6E−03^a^
IgG index	0.5 (0.3–0.6)	0.6 (0.4–1.1)	0.6 (0.3–2.7)	4.9E−03^a^
Q-Albumin	6.3 (3.5–13.3)	8.2 (3.6–25.3)	8.0 (1.9–136.9)	0.13^a^
Blood-CSF-barrier disruption [percent (count)]				
No	68.8 (11)	40 (4)	38.1 (8)	0.35^c^
Mild	31.3 (5)	50 (5)	42.9 (9)	
Moderate	0.0 (0)	0 (0)	4.8 (1)	
Severe	0.0 (0)	10 (1)	14.3 (3)	

^a^Kruskal-Wallis test

^b^χ^2^ test

^c^Fisher exact test

### Metabolite reprogramming between COVID-19 and viral CNS infections

**Fig. 1:**
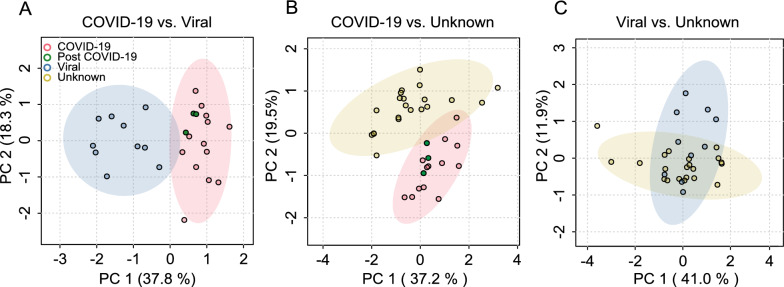
Cerebrospinal fluid (CSF) metabolite populations differ between COVID-19 with neurological involvement and non-COVID encephalitis/meningitis/myelitis. Principal component analysis (PCA) was performed based on 32 metabolites (detailed in Table S2) in the comparison between COVID-19 and viral central nervous system (CNS) infections [dCtrl (viral)] and clinical encephalitis/meningitis/myelitis without pathogen detection [dCtrl (unknown)], respectively. The y-axis label of A applies also to B and C. **A** COVID-19 vs. dCtrl (viral). **B** COVID-19 vs. dCtrl (unknown). **C** dCtrl (viral) vs. dCtrl (unknown). A PCA comprising all three groups is shown as Figure S1

**Fig. 2:**
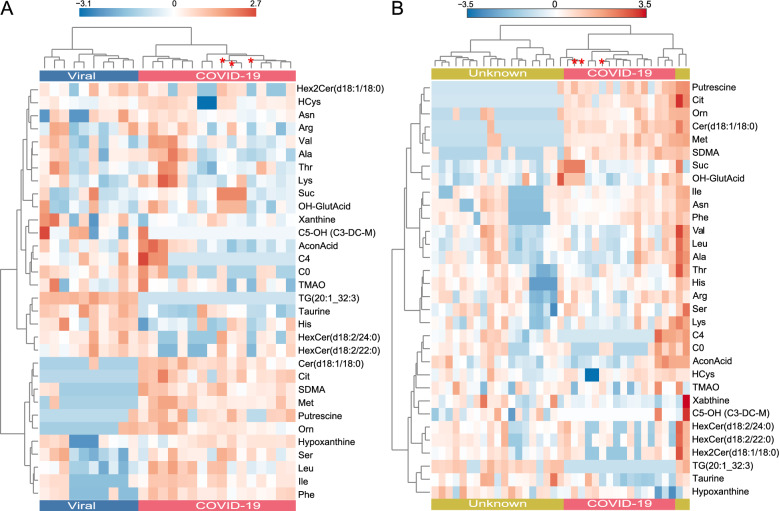
Classification of the cerebrospinal fluid (CSF) samples by unsupervised hierarchical clustering analysis. Analysis based on the same metabolite concentration data as used for the principal component analysis (PCA) in Fig. 1. The samples are organized along the x-axis, the diagnostic groups are indicated by a color code in the legend. The metabolites are clustered along the y-axis. Each colored cell on the map corresponds to the relative concentration of the analyte with respect to the mean-centered and divided by standard deviation of the analyte (z-score). **A** COVID-19 vs. dCtrl (viral). **B** COVID-19 vs. dCtrl (unknown). A clustering analysis based on mean metabolite concentrations per group is shown as Figure S3

A PCA based on the CSF metabolite data revealed clear differentiation between COVID-19 and the other two groups, whereas there was very little separation between the two non-COVID groups (Fig. 1). The top two metabolites driving the first dimension, which contributed most (37.8% and 37.2%) to variation, are one triglyceride (TG[20:1_32:3]) and symmetric dimethylarginine (SDMA) for the comparison COVID-19 vs. dCtrl (viral) and ornithine (Orn) and ceramide (Cer[d18:1/18:0]) when comparing COVID-19 vs. dCtrl (unknown) (Figure S2). The second dimension was predominantly influenced by concentration changes in trimethylamine N-oxide (TMAO) and acylcarnitines C0 and C4 in the comparison COVID-19 vs. dCtrl (viral) and by TG(20:1_32:3) and Cer(d18:1/18:0) in the comparison COVID-19 vs. dCtrl (unknown). One of the three post-COVID-19 samples (ID 57690) localized to the intersects between the COVID-19 and non-COVID samples, whereas the two other post-COVID-19 samples localized in the center of the CI of the COVID-19 samples. In an unsupervised hierarchical clustering analysis based on mean metabolite concentrations, dCtrl (viral) and dCtrl (unknown) clustered together in one clade, and in most metabolites the direction of concentration change with respect to COVID-19 was the same in dCtrl (viral) and dCtrl (unknown) (Figure S3). Next, we performed two-group hierarchical clustering analyses based on metabolite concentrations in each sample. All dCtrl (viral) samples could be separated perfectly from COVID-19, whereas one of the dCtrl (unknown) samples clustered with the COVID-19 samples (Fig. 2). In both two-group clustering analyses, two of the three post-COVID-19 samples were found to form a small subclade, but overall agreed with the PCA in that they did not constitute a distinct disease subtype with respect to CSF metabolites. Thus, both the PCA and hierarchical clustering analyses demonstrated that the disease controls were much more similar to each other than to COVID-19.

### Biomarker screening

Since the PCA and hierarchical clustering analysis suggested that there are meaningful differences in CSF metabolite abundance patterns between neuro-COVID and the two non-COVID groups, we performed a differential abundance analysis (Figure S4). Indeed, significant differences in concentrations of individual metabolites were observed. 11 and 7 analytes were significantly (*p* < 0.05, FDR) differentially abundant with respect to dCtrl (viral) and dCtrl (unknown), respectively. Specifically, 9 and 5 analytes were higher in dCtrl (viral) and dCtrl (unknown), respectively, and 2 were lower in both. Of note, all 7 differentially abundant metabolites in dCtrl (unknkown) are among the 11 differentially abundant metabolites in dCtrl (viral): one triglyceride (TG[20:1_32:3]) and the nonstructural one amino acid taurine were lower, whereas one amino acid [methionine (Met)], two amino acid metabolites [ornithine (Orn), SDMA], one biogenic amine (putrescine), and Cer(d18:1/18:0) were higher in COVID-19 than in both non-COVID groups.

**Fig. 3:**
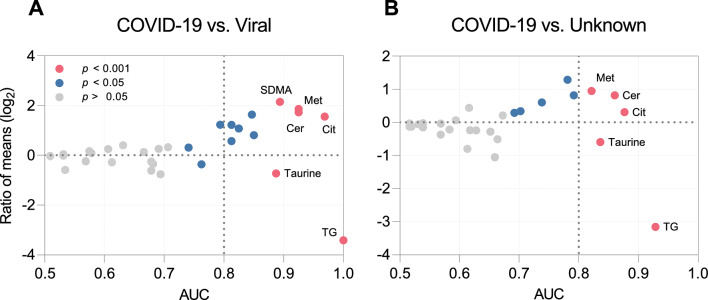
Identification of cerebrospinal fluid (CSF) metabolite biomarkers by receiver operating characteristic (ROC) curve analysis. Dispersion plots based on the same CSF metabolites as used for the principal component analysis (PCA) and clustering analyses shown in Figs. 1 and 2. The y-axis represents the ratio of mean concentrations in COVID-19/dCtrl (viral) or dCtrl (unknown). Area under the ROC curve (AUC) values are plotted along the x-axis. Each circle represents one metabolite, and the fill color indicates the asymptotic significance of the ROC curve. The y-axis label of A also applies to B. **A** COVID-19/dCtrl (viral). **B** COVID-19/dCtrl (unknown)

We then applied ROC curve analysis to identify accurate biomarkers, as defined by an AUC ≥ 0.8 (corresponding to excellent classification [28]), lower CI > 0.5, and asymptotic *p* ≤ 0.05. We first performed a high AUC abundance curve analysis (HAUCA) analysis to estimate the risk of false positive discovery (Figure S5). This risk turned out to be very low, as the likelihood of identifying a biomarker with an AUC ≥ 0.8 by chance alone was 0.1% (CI 1%) and 0% (CI 0%) for COVID-19 vs. dCtrl (viral) and COVID-19 vs. dCtrl (unknown), respectively. The dispersion plots in Fig. 3 illustrate that 11 and 5 analytes satisfied the above three criteria for the differentiation of COVID-19 from dCtrl (viral) and dCtrl (unknown), respectively, whereas there was only one for the differentiation between dCtrl (viral) and dCtrl (unknown) (Figure S6). Table 2 summarizes the AUCs of standard CSF parameters and metabolite biomarkers. TG(20:1_32:3) proved to be a perfect biomarker (AUC = 1.0) to differentiate COVID-19 from dCtrl (viral). The differentiation from dCtrl (unknown) was less perfect, but TG(20:1_32:3) was also the most accurate biomarkers for this comparison. Of note, all metabolites [except C4 in dCtrl (viral) and citrulline (Cit) in dCtrl (unknown)] had also passed the cut-off in the differential abundance analysis (Figure S4), providing additional internal validation for these markers (marked with * in Table 2). Internal cross-validation using the jackknife method confirmed that TG(20:1_32:3) was among the most robust metabolite biomarkers, as it consistently ranked among the top 5 biomarkers. Consistent with the large differences in neuroinflammation between COVID-19 and the disease control groups seen in Table 1, the diagnostic potential of standard CSF parameters was high. The standard CSF parameter with the highest accuracy to differentiate COVID-19 from both control groups was cell count (AUC = 0.99, 0.98), followed by protein concentration and IgG index. Of the peripheral blood parameters, only CRP had some value, i.e. AUC = 0.77 for the differentiation between COVID-19 and dCtrl (viral).

**Table 2:** Comparison of standard CSF and blood parameters and potential CSF metabolite biomarkers to differentiate between COVID-19 and disease control group by receiver operating characteristic curve analysis

Standard CSF and blood parameters		CSF metabolites			
Parameter	AUC (CI)	Metabolite	AUC (CI)	Frequency of selection in leave-one-out cross-validation^a^	
				Top 5	Top 10
**COVID-19 vs disease Ctrl (viral)**					
CSF cell count	0.99 (0.96–1.00)	TG(20:1_32:3)*	1.00 (1.00–1.00)	1	1
CSF protein	0.90 (0.75–1.00)	Cit*	0.97 (0.91–1.00)	1	1
IgG index	0.84 (0.63–0.97)	Cer(d18:1/18:0)*	0.93 (0.78–1.00)	1	1
Q-albumin	0.65 (0.41–0.86)	Met*	0.93 (0.78–1.00)	1	1
CSF lactate	0.53 (0.26–0.70)	SDMA*	0.90 (0.75–1.00)	0.70	1
Blood CRP	0.77 (0.58–0.93)	Taurine*	0.89 (0.75–1.00)	0.19	1
Blood leukocytes	0.58 (0.35–0.79)	Leu*	0.85 (0.69–0.98)	0.04	1
		Putrescine*	0.85 (0.69–1.00	0.04	1
		Orn*	0.83 (0.59–1.00)	0.04	0.81
		C4	0.81 (0.61–1.00)	0	0.58
		Ile*	0.81 (0.63–0.99)	0	0.77
**COVID-19 vs disease Ctrl (unknown)**					
CSF cell Count	0.98 (0.90–1.00)	TG(20:1_32:3)*	0.93 (0.85–1.00)	1	1
CSF protein	0.78 (0.62–0.91)	Cit	0.88 (0.74–1.00)	1	1
IgG index	0.82 (0.66–0-93)	Cer(d18:1/18:0)*	0.86 (0.71–1.00)	1	1
Q-albumin	0.67 (0.52–0.86)	Taurine*	0.84 (0.70–0.98)	1	1
CSF lactate	0.51 (0.31–0.67)	Met*	0.82 (0.66–0.98)	0.97	1
Blood leukocytes	0.67 (0.48–0.85)				
Blood CRP	0.64 (0.46–0.83)				

AUC: area under the curve; CI: confidence interval; CRP: C-reactive protein

^a^1: always selected; 0: never selected

**Fig. 4:**
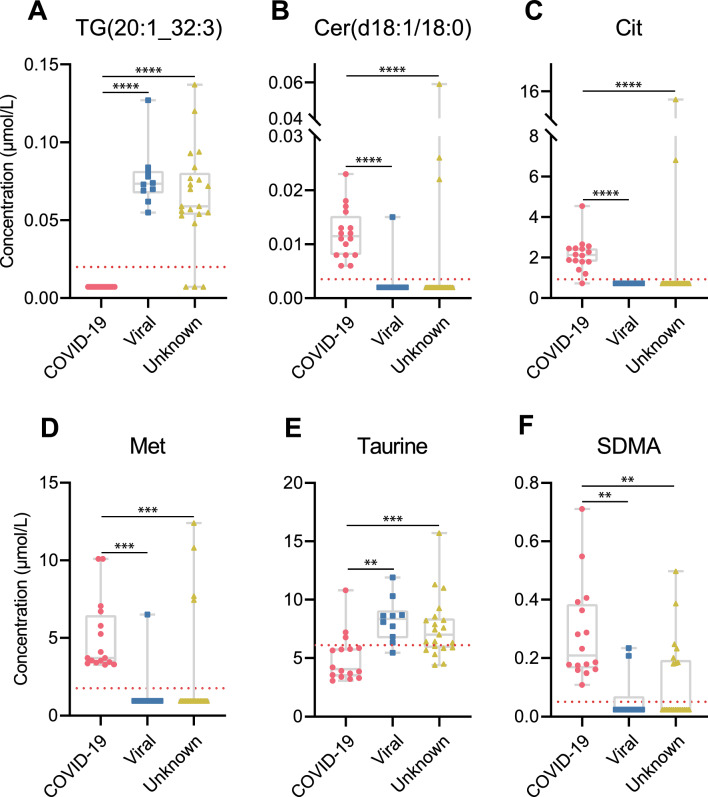
Concentrations of the six best-validated metabolite biomarkers for the discrimination between COVID-19 with neurological involvement and dCtrl (viral). They also include the best five for the discrimination between COVID-19 and dCtrl (unknown). The red dotted line represents optimal cut-off values in the ROC curve, indicating the optimal trade-off between sensitivity and specificity, generated by the Youden index method. The y-axis labels of A and D also apply to B, C, E, F. **A** TG (20:1_32:2); **B** Cer(d18:1/18:0); **C** Cit; **D** Met; **E** Taurine; **F** SDMA. Significance of between-group differences is indicated as **p < 0.01, ***p < 0.001, ****p < 0.0001 (Mann–Whitney U-test)

In Table 3 we then assessed parameters that are routinely used to evaluate diagnostic tests, i.e. sensitivity, specificity, PPV, and NPV of the top 3 standard parameters (based on AUC) and the best validated biomarkers, as defined by fulfilling all of the following criteria: (i) AUC ≥ 0.8, lower CI ≥ 0.5, asymptotic *p* ≤ 0.05 in ROC analysis, (ii) 100% selection frequency among the top 10 markers in leave-one-out cross-validation, and (iii) FC ≥|1.5| and FDR ≤ 0.05 in differential abundance analysis (Figure S4). Four of the best validated biomarkers (Fig. 4) showed excellent discriminatory power between COVID-19 and dCtrl (viral), exhibiting high Youden index values (defined as sensitivity + specificity−1) of ≥ 0.90, indicating both high sensitivity and specificity. Particularly noteworthy is TG(20:1_32:3), which surpasses all other standard parameters and biomarkers, demonstrating perfect sensitivity and specificity of 1. Notably, among the standard parameters, CSF cell count emerged as the sole parameter with a similarly high Youden index (0.94), while CSF protein or IgG index displayed either low sensitivity or specificity. In differentiating between COVID-19 and dCtrl (unknown), CSF cell count exhibited the highest Youden index (0.94), followed by the biomarkers TG(20:1_32:3) and Cer(d18:1/18:0) (0.86). It is worth mentioning that these biomarkers demonstrate slightly higher specificity than CSF cell count. Figure 4 presents the concentrations of the six best validated biomarkers in the differentiation between COVID-19 and dCtrl (viral) across all three groups. They also include the best five for the discrimination between COVID-19 and dCtrl (unknown). Specifically, concentrations of TG(20:1_32:3) are consistently < LOD in the COVID-19 samples, contrasting with values > LOD in nearly all disease control cases. The red dotted line signifies optimal cut-off values derived from the ROC curve, indicating the optimal balance between sensitivity and specificity, determined by the Youden index method. Notably, a cut-off value of 0.02 [µmol/L] effectively distinguishes between all COVID-19 cases and viral disease controls [dCtrl (viral)]. In contrast to the low levels of TG(20:1_32:3) and taurine in the COVID-19 samples, concentrations of other well-validated biomarkers (Cer(d18:1/18:0), Met, Cit and SDMA) are predominantly > LOD in this group but primarily below LOD in the control groups.

**Table 3:** Comparison of diagnostic accuracy between CSF cell count, CSF protein concentration, IgG index, and the best validated CSF metabolite biomarkers^a^

Parameter^b^	Sensitivity	Specificity	PPV	NPV	Cut-off value	Youden index^c^
COVID-19 vs. disease Ctrl (viral)						
TG(20:1_32:3)	1.00	1.00	1.00	1.00	0.02 [µmol/L]	1.00
CSF cell count	1.00	0.94	0.96	1.00	5.30 [cells/µL]	0.94
Cit	1.00	0.94	0.96	1.00	0.93 [µmol/L]	0.94
Cer(d18:1/18:0)	0.90	1.00	1.00	0.86	0.0035 [µmol/L]	0.90
Met	0.90	1.00	1.00	0.86	1.77 [µmol/L]	0.90
CSF protein	1.00	0.81	0.89	1.00	0.42 [g/L]	0.81
SDMA	0.80	1.00	1.00	0.76	0.051 [µmol/L]	0.80
Taurine	0.90	0.81	0.88	0.84	6.1 [µmol/L]	0.71
Peutrescine	0.90	0.81	0.88	0.84	0.0058 [µmol/L]	0.71
Leu	0.70	0.94	0.95	0.66	9.9 [µmol/L]	0.64
IgG index	0.70	0.88	0.9	0.65	0.57	0.58
COVID-19 vs. disease Ctrl (unknown)						
CSF cell count	1.00	0.94	0.93	1.00	3.03 [cells/µL]	0.94
TG(20:1_32:3)	0.86	1.00	1.00	0.90	0.02 [µmol/L]	0.86
Cer(d18:1/18:0)	0.86	1.00	1.99	0.90	0.0035 [µmol/L]	0.86
Met	0.81	1.00	1.00	0.87	1.77 [µmol/L]	0.81
CSF protein	0.76	0.81	0.76	0.82	0.42 [g/L]	0.57
Taurine	0.76	0.81	0.76	0.82	5.9 [µmol/L]	0.57
IgG index	1.00	0.56	0.66	1.00	0.47	0.56

^a^Best validated CSF metabolite biomarkers, as defined by meeting all of the following criteria: ROC analysis: AUC ≥ 0.8, lower CI 0.5, asymptotic *p* ≤ 0.05 (Table 2). Leave-one-out cross-validation: 100% selection frequency among the top 10 markers (Table 2). Differential abundance analysis: fold change ≥|1.5|, FDR ≤ 0.05 (Figure S4)

^b^Ranked by Youden index in descending order

^c^Sensitivity + specificity−1

### Correlation of metabolites with markers of systemic and CNS inflammation

**Fig. 5:**
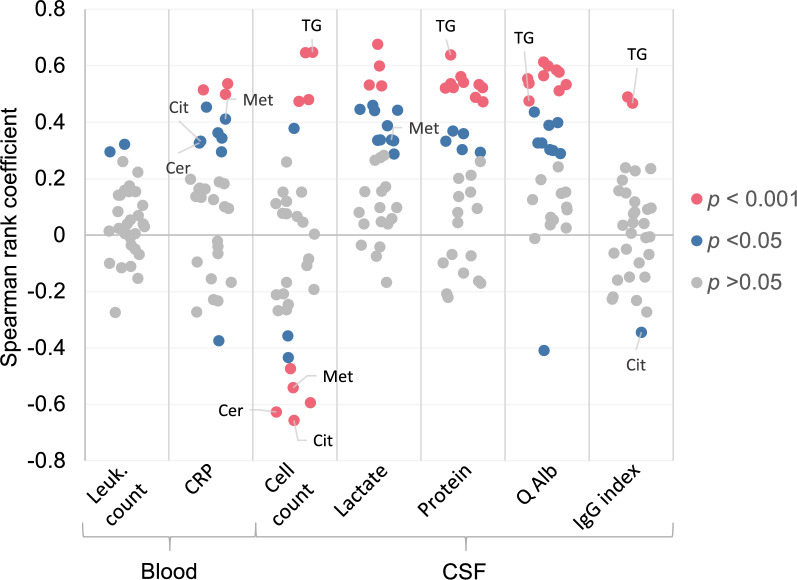
Correlation analysis between metabolite concentrations and standard blood and cerebrospinal fluid (CSF) parameters across all samples. Correlation is based on the same CSF metabolites as used for the analyses shown in Figs. 1 and 2. The y-axis represents the Spearman rank correlation coefficient. Each circle represents a specific metabolite, and the fill color indicates significance of correlation. The labelled metabolites are the best validated biomarkers with a Youden index ≥ 0.9 (Table 3). TG: triglyceride; Cer: ceramide; Met: methionine, Cit: citrulline

We then investigated to what extent the observed changes in metabolite concentrations were driven by inflammation in CNS. To this end, we determined the degree of correlation, across all samples, between CSF metabolite concentrations and parameters that indicate inflammation in CNS or peripheral blood (Fig. 5). Correlations were generally positive, with the exception of CSF cell count, which also featured moderate negative correlations. Particularly significant and robust correlations were observed with inflammation markers in CSF, namely cell count, lactate, total protein, and Q-albumin. A reciprocal relationship was noted between blood CRP and CSF cell count in that some of the same metabolites that correlated positively with CRP correlated negatively with CSF cell count. The best validated biomarkers are highlighted in the plot. Notably, TG(20:1_32:3) correlated moderately positively with parameters of CSF inflammation, suggesting that alterations in TG(20:1_32:3) primarily reflect local CNS pathology rather than systemic manifestations. However, the relatively modest correlation coefficients suggested that the higher levels in the dCtrl (viral) and (unknown) samples are only partially driven by the more pronounced neuroinflammation in these samples. Interestingly, Met, Cer(d18:1/18:0), and Cit correlated negatively with CSF cell count but positively with peripheral blood CRP. Taken together, these results suggest that the observed differences in metabolite concentrations between the COVID-19 and the non-COVID samples are partially driven by differences in neuroinflammation, but other mechanisms likely contribute.

### Lack of impact of immunosuppressive therapy on the CSF metabolites

**Fig. 6:**
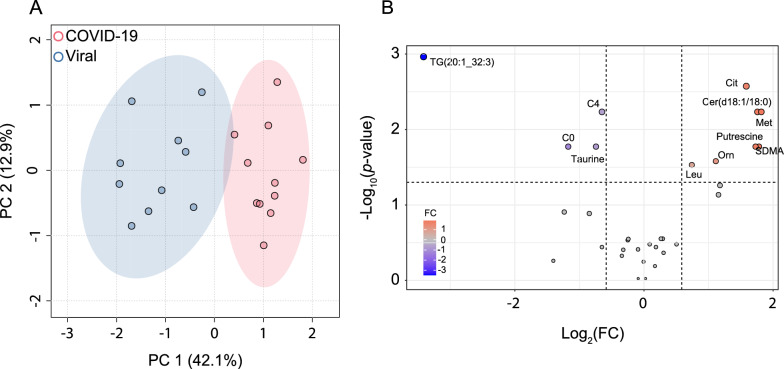
Immunosuppressive therapy has only a minor effect on the cerebrospinal fluid (CSF) metabolome. **A** Principal component analysis (PCA) between the COVID-19 samples without immunosuppressive therapy and dCtrl (viral). The PCA was performed based on the same metabolites as in Fig. 1. **B** Differential abundance analysis based on the same data set as the PCA. The ratio of mean concentration (“fold change (FC)”, COVID-19/controls) is plotted log_2_ transformed on the y-axis, adjusted *p* value log_10_ transformed (corrected for multiple testing by Benjamini–Hochberg correction) on the x-axis. The threshold was set to FC ≥|1.5| and the adjusted *p* < 0.05. TG: triglyceride; Cer: ceramide; Met: methionine; C0 (carnitine) and C4 (butyrylcarnitine): acylcarnitines; Leu: leucine; Orn: ornithine; Met: methionine; Cit: citrulline; SDMA: symmetrical dimethylarginine

31.2% of COVID-19 and 23.8% of dCtrl (unknown), but none of the dCtrl (viral) patients were treated with immunosuppressive therapy at the time of lumbar puncture (*p* = 0.16, Fisher’s exact test). The influence of immunosuppressive therapy, particularly with glucocorticoids, on patient metabolism is widely acknowledged [29]. A two-group comparison of CSF metabolite concentrations between COVID-19 cases with and without immunosuppressive therapy revealed minimal differences between the groups in a PCA (Figure S7), and a differential abundance analysis did not identify any significantly altered metabolites (Figure S8). Additionally, a PCA conducted between COVID-19 cases without immunosuppressive therapy and dCtrl (viral) (Fig. 6A) exhibited a similar separation pattern compared to the PCA between all COVID-19 samples and dCtrl (viral) (Fig. 1). Moreover, the differential abundance analysis identified the same 10 metabolites (along with acylcarnitines C0 and C4) (Fig. 6B) that had been identified in the analysis involving all COVID-19 samples (Figure S4A). These findings ruled out that the neuro-COVID-associated CSF metabolite biomarkers identified in our study resulted from confounding effects from immunosuppressive therapy.

## Discussion

We conducted a targeted metabolic analysis on CSF samples obtained from COVID-19 patients exhibiting neurological manifestations in both acute and post-COVID-19 stages. Comparative assessments were made against two disease control groups with CNS infections (encephalitis/meningitis/myelitis): (i) with confirmed viral pathogen [dCtrl (viral)] and (ii) without identification of a pathogen [dCtrl (unknown)]. Our current study confirmed the discriminatory potential of CSF metabolites in CNS infections and revealed distinct metabolic profiles, facilitating robust differentiation between the groups based on metabolic alterations. Particularly noteworthy was the high accuracy of triglyceride TG[20:1_32:3], which surpassed all conventional parameters and other metabolite biomarkers. Its remarkable discriminatory ability stemmed from the fact that it was lower in COVID-19 than in all dCtrl (viral) and most dCtrl (unknown) samples. Considering its moderate positive correlation with indices of CNS inflammation and BCB disruption, the elevated levels in the disease controls are partially driven by ongoing neuroinflammation (which is much lower in COVID-19 samples). However, in the absence of CSF samples from healthy individuals as reference we cannot rule out that a reduction in levels of this triglyceride in CSF is part of the pathology of neuro-COVID.

Triglycerides generally serve as an energy source, contribute to the structural integrity and stability of cellular membranes, are activators of cell signaling pathways [30] and are also closely related to inflammatory processes in the CNS [31]. Contrary to the low levels of TG(20:1_32:3) (< LOD) in the CNS, this specific TG was significantly up-regulated in serum from patients with COVID-19 and serves as a biomarker to differentiate COVID-19 from controls [32]. Considering the high degree of systemic inflammation in COVID-19, the high levels of this TG in peripheral blood are likely driven by systemic inflammation, whereas the low levels in CSF are consistent with the low degree of classic neuroinflammation in CSF in neuro-COVID. TG(20:1_32:3) has six potential isomers, therefore the significance of its differential abundant concentration is uncertain and we can only speculate on role in pathophysiology. Possible hydrolysis products of TG(20:1_32:3) are myristic acid, lauric acid, and myristoleic acid. Only little is known about these specific fatty acids in neuroinflammatory diseases, but myristic acid plays a crucial role as a metabolic checkpoint in maintaining immune homeostasis by regulating the stimulation of interferon gamma-dependent autophagy that is especially seen in HSV-1 infection [33].

Ceramide Cer(d18:1/18:0) was the second-best biomarker for the differentiation between neuro-COVID and both control groups. Ceramides are highly lipophilic and form large rafts in plasma membrane that are used by SARS-CoV-2 for cell entry [34]. However, the elevated ceramide concentration in CSF is unlikely due to an interaction of CNS cells with SARS-CoV-2, as the virus is usually not detected in CNS, or only at very low levels. Higher levels of Cer(d18:1/18:0) in the neuro-COVID samples might be partially due to higher activity of acid sphingomyelinase that cleaves membrane-bound or free sphingomyelins into ceramides [35]. Alternatively, it could be due to lower activity of this enzyme in the disease control groups. Clearly, the functional significance of the elevated Cer(d18:1/18:0) levels in neuro-COVID CSF is uncertain and requires further studies.

We detected higher levels of the short-chain acylcarnitine C4 in dCtrl (viral) than in COVID-19 (e.g., Figure S3), which also qualified as accurate biomarkers (Table 2). This result agrees well with our findings in an independent cohort, that short-chain acylcarnitines C4 and C5 are elevated in CSF from patients viral CNS infections, presumably because of dysfunctional fatty acid oxidation (β-oxidation) [19].

From a clinical perspective, it is worth mentioning that our study revealed CSF cell count as the best standard parameter to differentiate between COVID-19 and the two diseased control groups. This finding confirms the value of CSF cell count as a reliable biomarker in routine CSF analysis to distinguish between neuro-COVID and aseptic meningitis/encephalitis. Although the top two metabolite biomarkers (TG[20:1_32:3] and citrulline) exhibit similar sensitivity and negative predictive value as CSF cell count, they exhibit superior specificity and positive predictive values. These properties are crucial in reducing the occurrence of false positive test results, which, in turn, helps prevent unnecessary treatment and interventions.

This study is limited by the small sample size in the dCtrl (viral) group and the small number of metabolites included in the analyses. Internal cross-validation by the jackknife method showed that the likelihood of false positive findings was very low, but future studies should be directed at validating the current findings in independently recruited cohorts. In this regard, it should be noted that metabolite biomarkers that were discovered in small cohorts, including those analyzed with Biocrates kits, may show lower accuracy when measured in an independent cohort [36]. Furthermore, the pathophysiological significance of the low levels of (TG[20:1_32:3] in the neuro-COVID samples remains uncertain due to two factors: (i) the absence of healthy controls for comparison, and (ii) the uncertainty surrounding the exact molecular identity of TG(20:1_32:3) (Table S2), as the Biocrates platform lacks specificity on double bond position, acyl length, and exact fatty acid makeup.

## Conclusion

Our findings highlight the importance of CSF metabolic profiling in identifying biomarkers for CNS infections. We have identified a limited number of CSF metabolite biomarkers that have the potential to differentiate between viral CNS infections caused by classic pathogens, aseptic encephalitis/meningitis/myelitis of unknown etiology, and neuro-COVID. Analysis based on standard parameters revealed that the COVID-19 samples exhibited significantly less neuroinflammation, but notably higher systemic inflammation. The observed metabolic alterations in CSF, combined with changes in standard CSF parameters, lend further support to the notion that neurological deficits in neuro-COVID patients are mediated by host-derived factors rather than viral replication in CNS. In particular, the reduced concentration of triglycerides (TG[20:1_32:3]) in CSF of patients with neuro-COVID proved to be an excellent biomarker, exceeding all standard parameters performance. Consequently, measuring these biomarkers in cerebrospinal fluid could offer additional diagnostic value and aid in risk stratification of patients suspected of having CNS infections.

### Supplementary Information

Supplementary Material 1.

## Data Availability

The dataset supporting the conclusions of this article is available in the Figshare repository at 10.6084/m9.figshare.25623534.v1.

## Abbreviations

AUC: Area under ROC curve
BCB: Blood-CSF-barrier
CI: Confidence interval
CNS: Central nervous system
COVID-19: Coronavirus disease 2019
CRP: C-reactive protein
CSF: Cerebrospinal fluid
dCtrl: Disease control
FC: Fold change
FDR: False discovery rate
FIA-MS/MS: Flow injection analysis tandem mass spectrometry
HAUCA: High AUC abundance
HSV: Herpes simplex virus
LC–MS/MS: Liquid chromatography-tandem mass spectrometry
LOD: Limit of detection
MHH: Hannover Medical School
NPV: Negative predictive value
PCA: Principal component analysis
PPV: Positive predictive value
ROC: Receiver operating characteristics
RT-PCR: Reverse transcriptase polymerase chain reaction
SARS-CoV-2: Severe acute respiratory syndrome coronavirus 2
VZV: Varicella zoster virus
χ^2^: Chi-squared test
