# Supplementary material for: Targeted metabolomics identifies accurate CSF metabolite biomarkers for the differentiation between COVID-19 with neurological involvement and CNS infections with neurotropic viral pathogens
Source: J Transl Med. 2024 Jul 3;22:620. doi: 10.1186/s12967-024-05422-1 (PMC11223383; doi:10.1186/s12967-024-05422-1)
Supplement: Supplementary file 1 — Supplementary Material 1. [file 12967_2024_5422_MOESM1_ESM.pdf]

# Supplemental material 1 = supplemental figures and tables

Neu F, et al. Targeted metabolomics identifies accurate CSF metabolite biomarkers for the differentiation between COVID-19 with neurological involvement and CNS infections with neurotropic viral pathogens. *J Transl Med* 2024

**Contents**

Figure S1.......................page 1

Figure S2.......................page 2

Figure S3.......................page 3

Figure S4.......................page 4

Figure S5.......................page 5

Figure S6.......................page 6

Figure S7.......................page 7

Figure S8.......................page 8

Table S1.......................page 9

Table S2.......................page 11

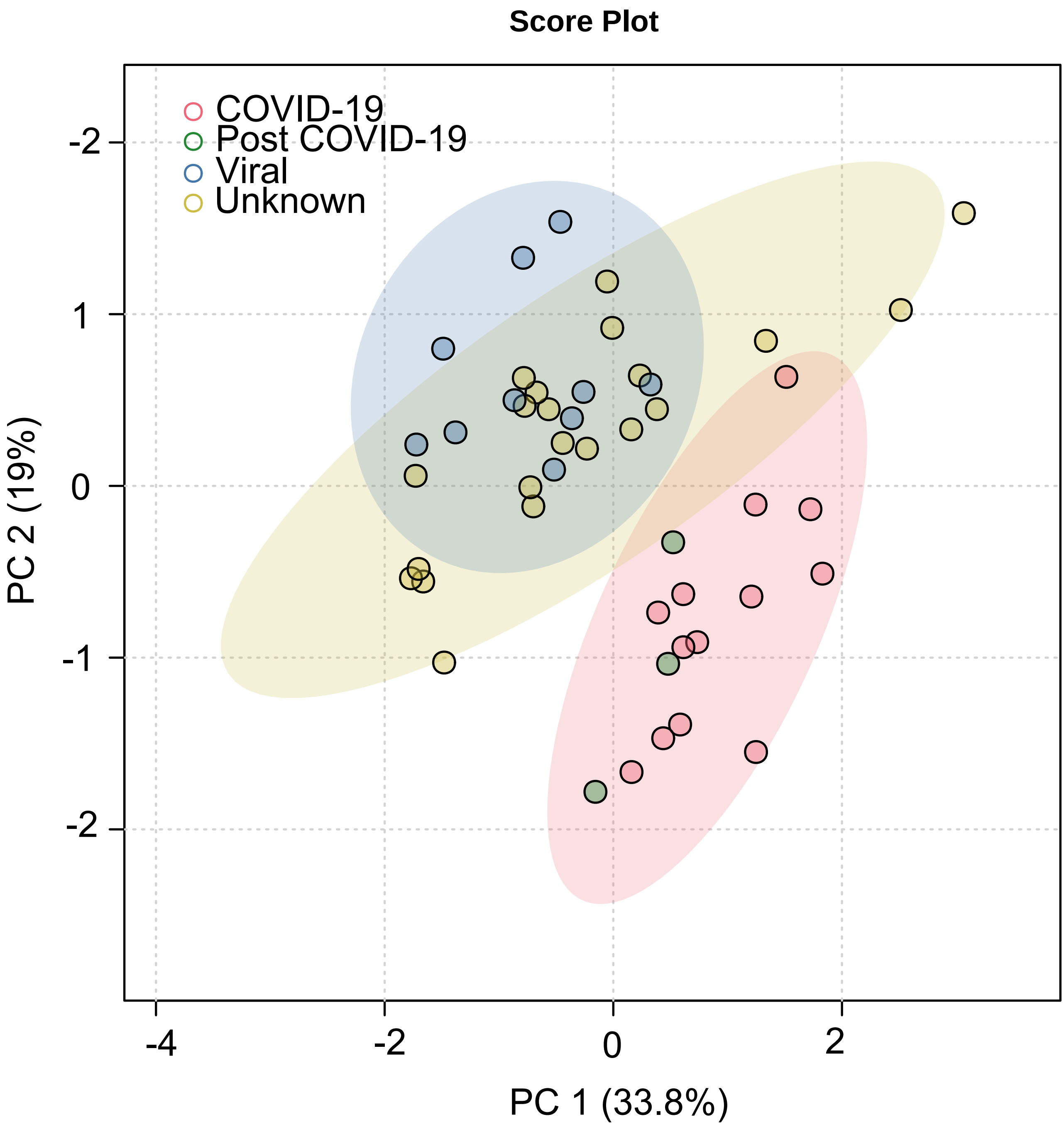


**Figure S1. Cerebrospinal fluid (CSF) metabolite populations differ between COVID-19 with neurological involvement and non-COVID encephalitis/meningitis/myelitis.** Principal component analysis (PCA) was performed based on 32 metabolites (detailed in Table S2) in the comparison between COVID-19 and viral central nervous system (CNS) infections (dCtrl [viral]) and clinical encephalitis/meningitis/myelitis without pathogen detection (dCtrl [unknown]), respectively.

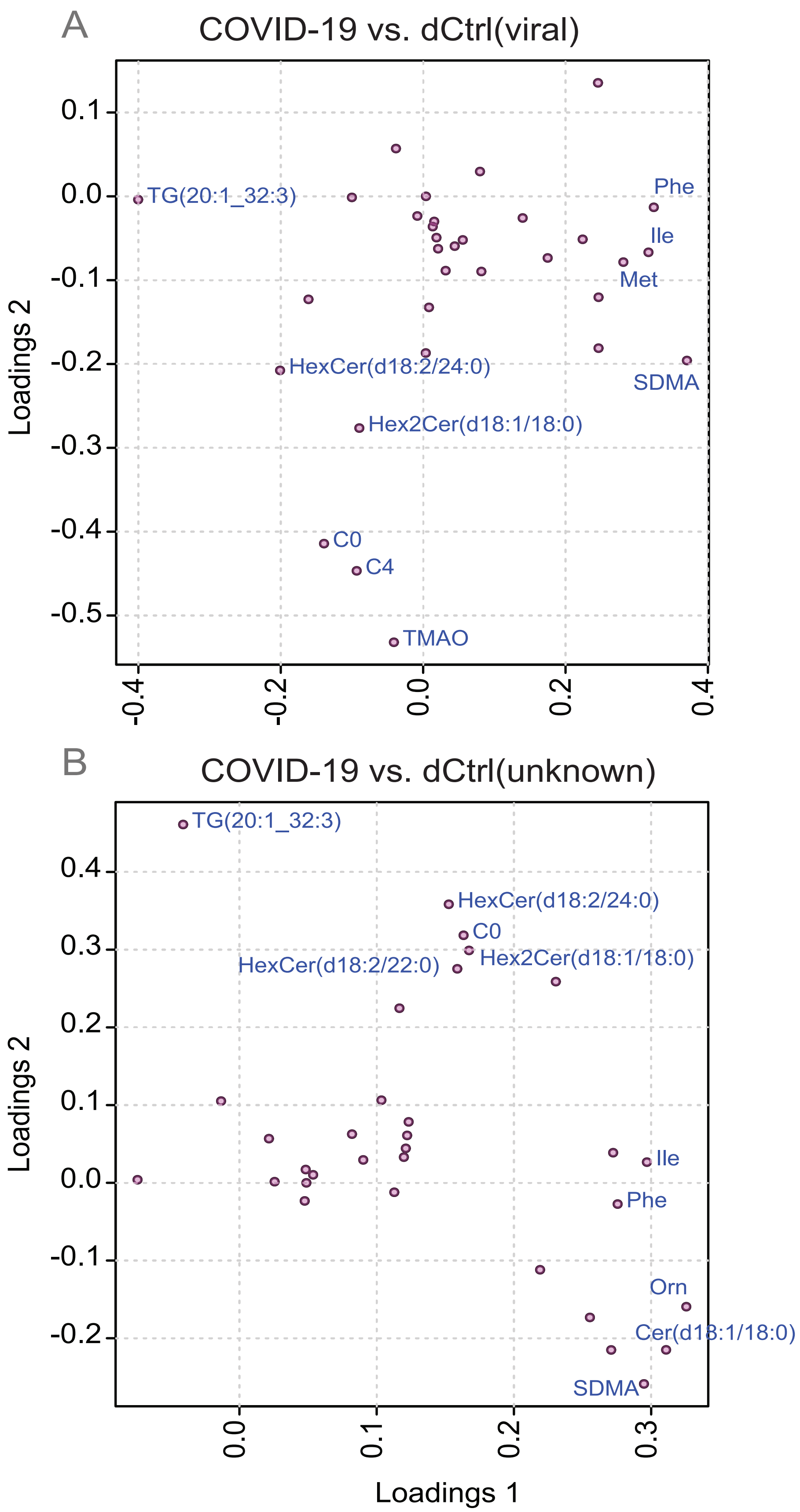


**Figure S2. Loading plots derived from principal component analyses (PCA) in Figure 1 depicting the influence of individual metabolites on the first two principal components (PC1 and PC2).** The x-axis delineates the contribution of each metabolite to PC1, while the y-axis delineates its contribution to PC2. The analysis is based on the 32 metabolites detailed in Table S2. The top five metabolites driving PC1 and PC2 are labeled in the figure. (**A**) COVID-19 vs. dCtrl (viral). (**B**) COVID-19 vs. dCtrl (unknown).

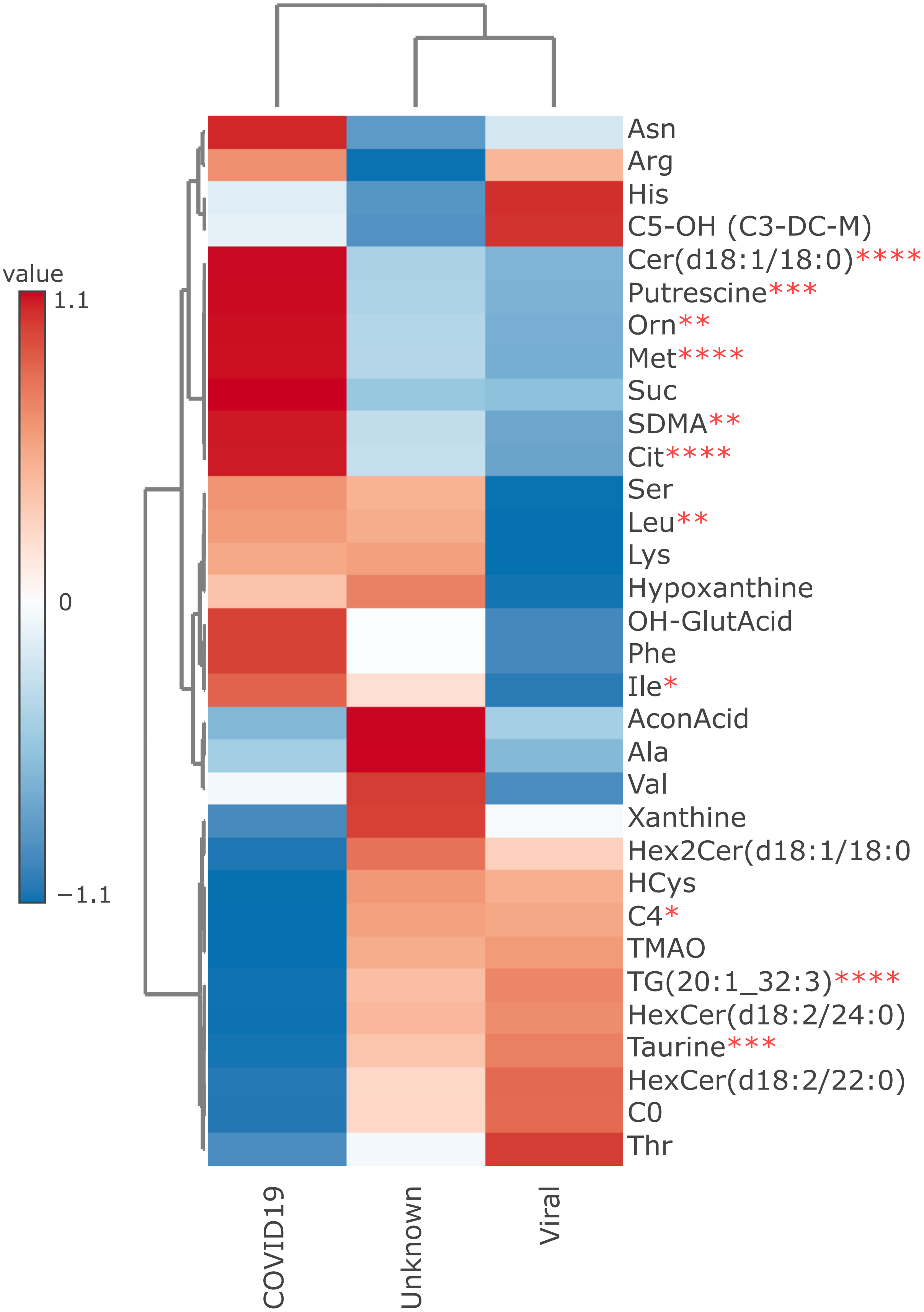


**Figure S3. Classification of the CSF metabolites and visualization of between-group differences by unsupervised hierarchical clustering analysis based on mean concentrations in each of the three groups.** Each colored cell in the heatmap corresponds to the group average concentration of the analyte with respect to the mean-centered and divided by standard deviation of the analyte (z-score). Y-axis = metabolite dendrogram. Significance of across-groups differences is indicated on the right; *$p < 0.05$, **$p < 0.01$, ***$p < 0.001$, ****$p < 0.0001$ (Kruskall-Wallis analysis).

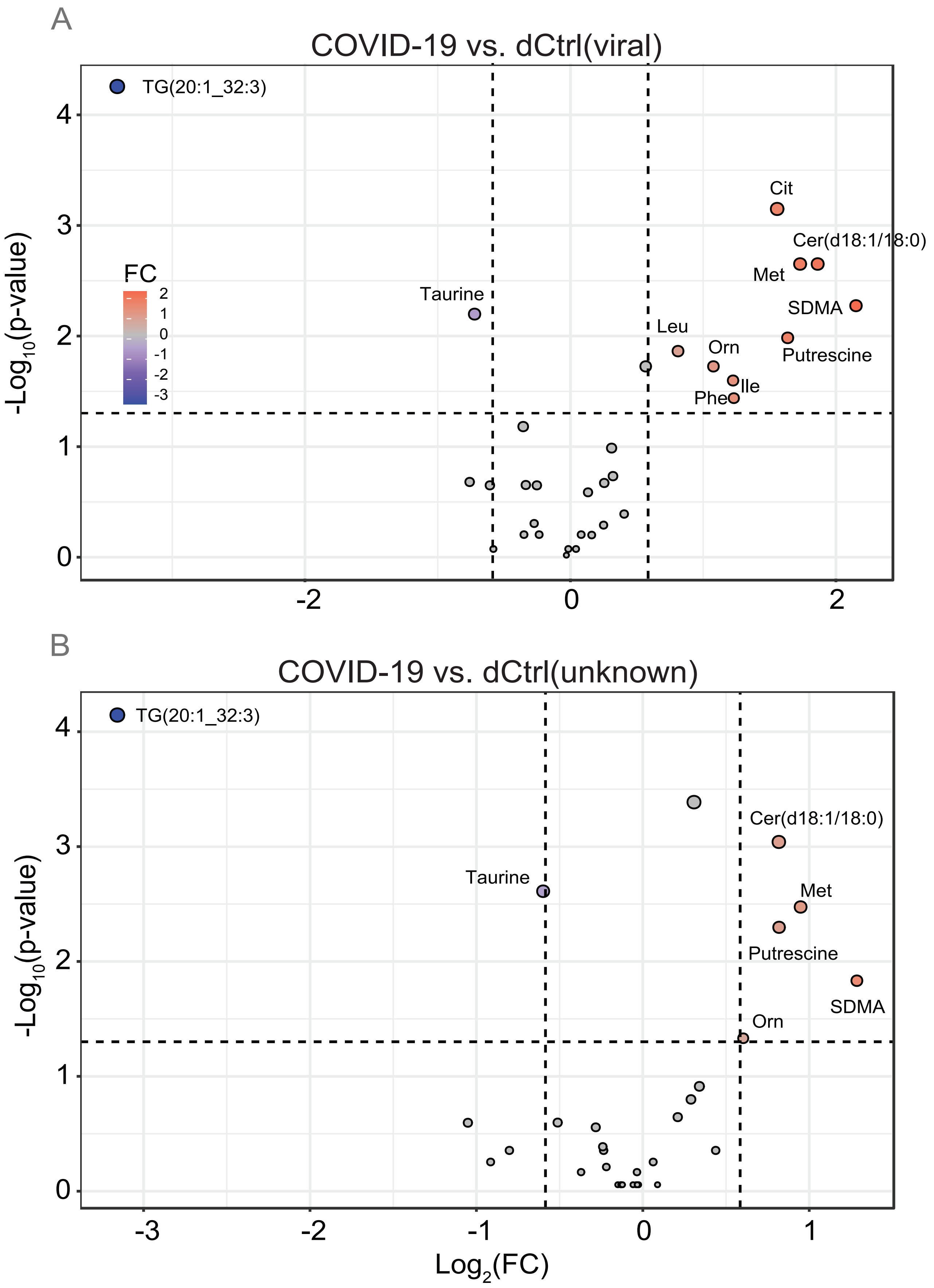


**Figure S4. Differential abundance analysis based on the same metabolites used in Figure 1.** The ratio of mean concentration ("fold change (FC)", COVID-19/controls) is plotted $\log_2$ transformed on the x-axis, adjusted *p* value $\log_{10}$ transformed (corrected for multiple testing after Benjamini-Hochberg) on the y-axis. The threshold was set to FC>|1.5| and the adjusted *p*-value <0.05. (**A**) COVID-19/dCtrl (viral). (**B**) COVID-19/dCtrl (unknown). Abbreviations: TG, triglyceride; Cer; ceramide; Met, methionine; Orn, ornithine; Phe, phenylalanine; Ile, isoleucine; Leu, leucine; Met, methionine; Cit, citrulline; SDMA, symmetrical dimethylarginine; Cer, ceramide.

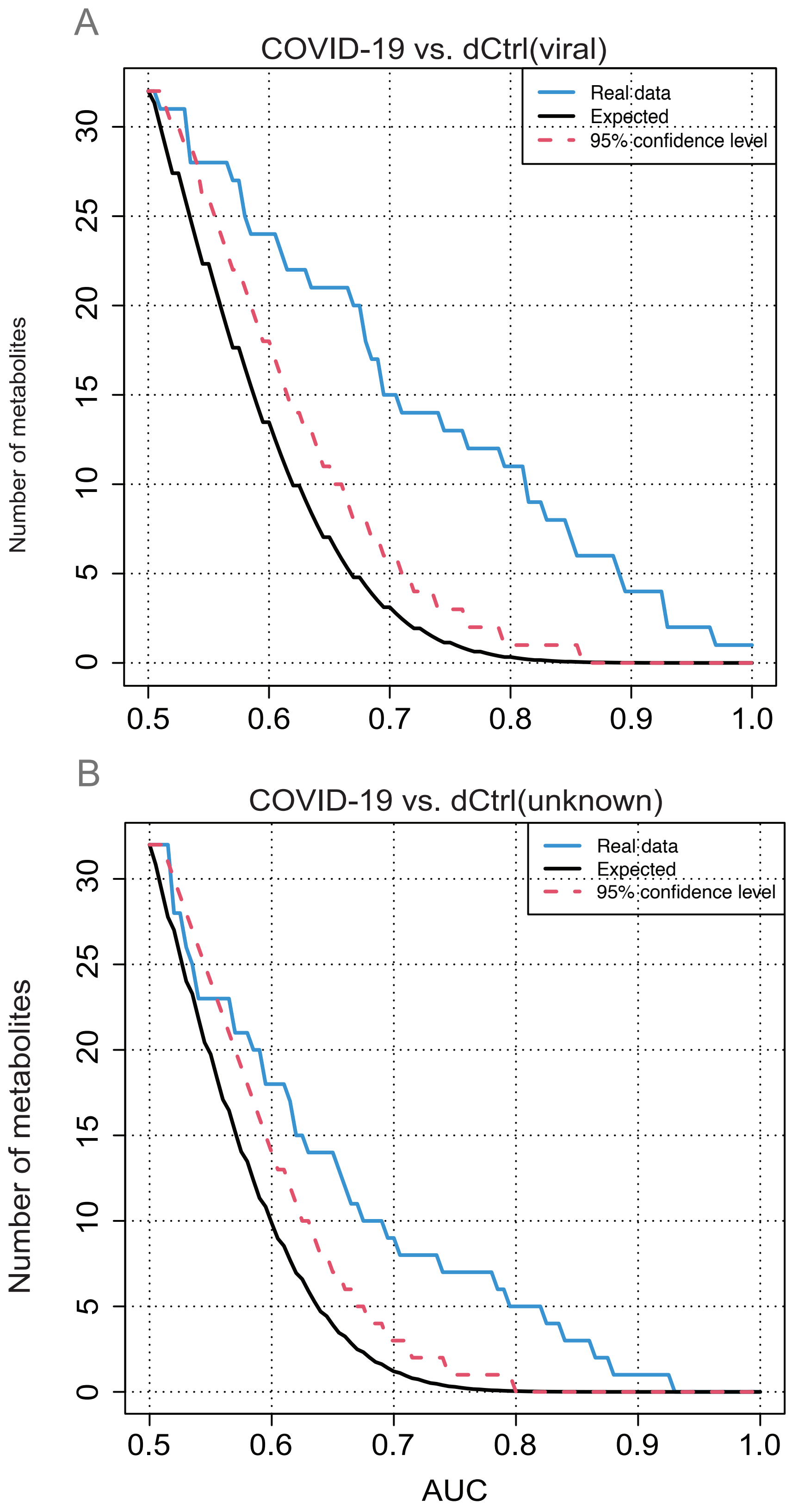


**Figure S5. High-throughput analysis of receiver operating characteristc curve (HAUCA) analysis to evaluate the likelihood of false positve biomarker identficaton.** The x-axis represents the area under the curve (AUC) threshold, while the y-axis illustrates the number of biomarkers. The analysis compares the number of biomarkers identified in the real data set (blue curve) to those expected in a random data set (black curve). The red dotted curve delineates the upper bound 95% confidence interval (CI) of the random data set. (**A**) COVID- 19 vs. dCtrl (viral). (**B**) COVID-19 vs. dCtrl (unknown). The likelihood of identifying a marker with AUC ≥ 0.8 in the random data set is 0.1% and 0% in A and B respectively.

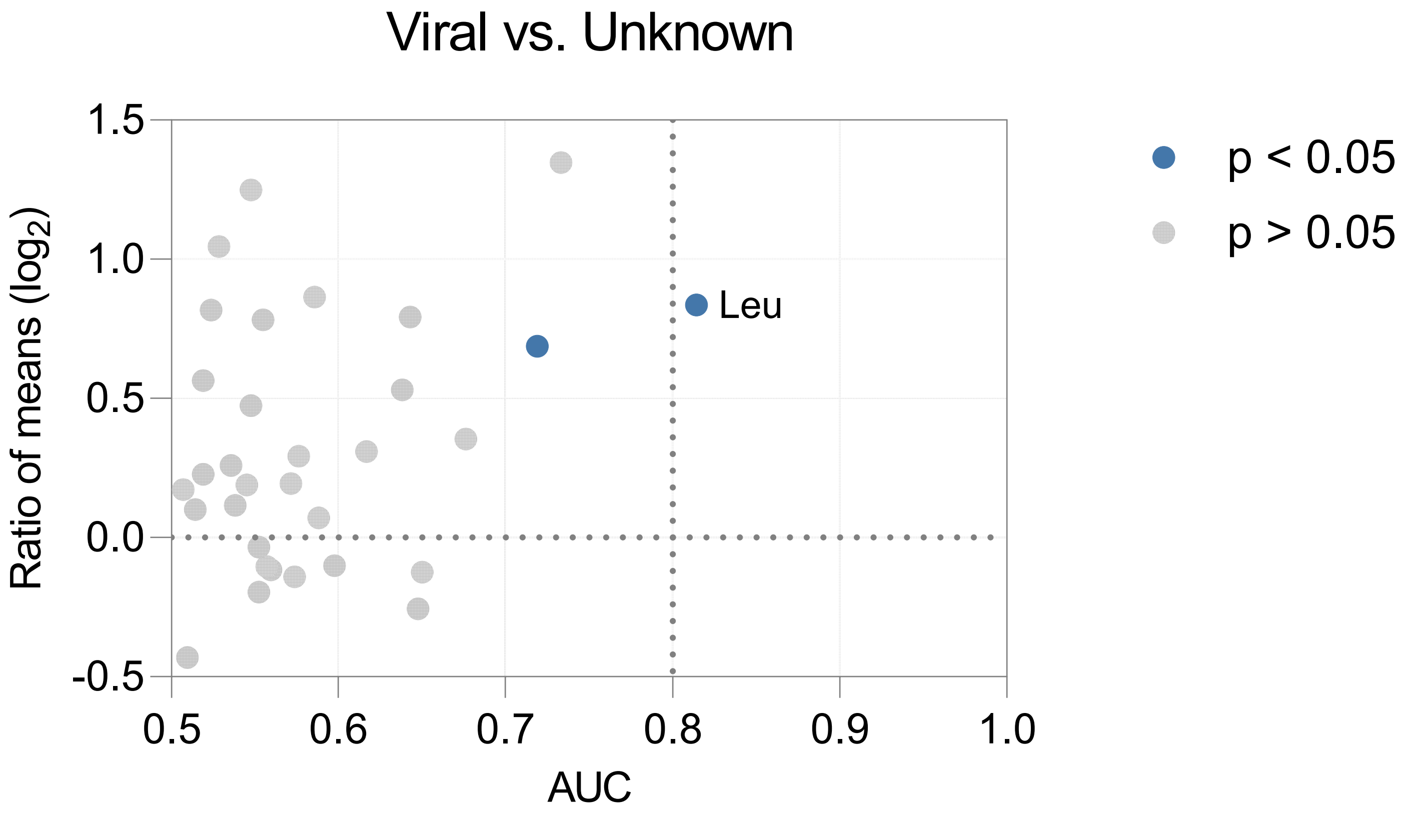


**Figure S6. Identification of CSF metabolite biomarkers by Receiver Operating Characteristic (ROC) curve analysis.** Dispersion plots based on the same CSF metabolites as used for the PCA and clustering analyses shown in Figure 1 and 2. The y-axis represents the ratio of mean concentrations in dCtrl (viral)/dCtrl (unknown). Area under the ROC curve (AUC) values are ploted along the x-axis. Each circle represents one metabolite, and the fill color indicates the significance of the ROC curve.

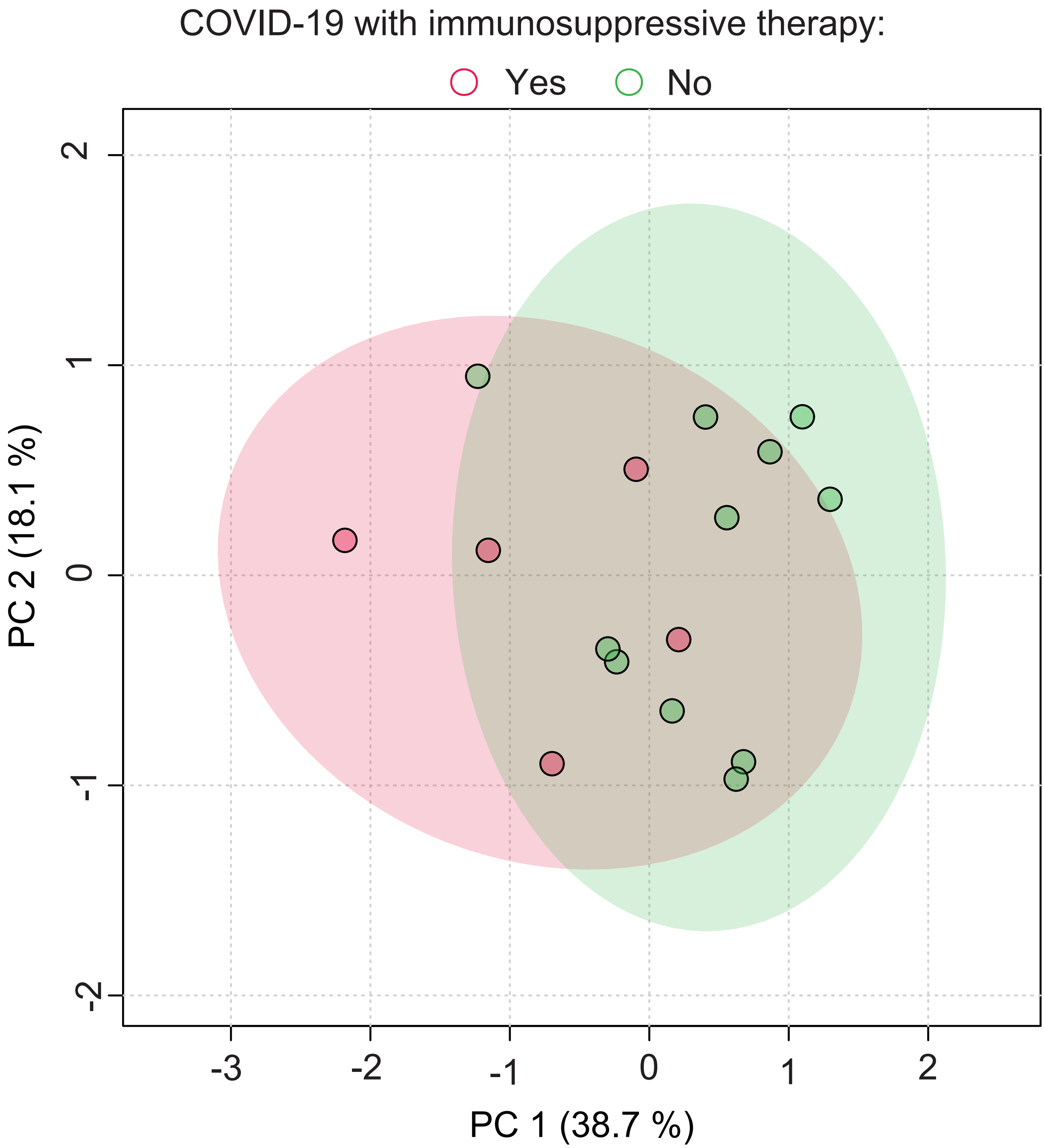


**Figure S7. Cerebrospinal fluid (CSF) metabolite populations do not differ significantly between COVID-19 with and without immunosuppressive therapy.** PCA was performed based on the same metabolites as in Figure 1.

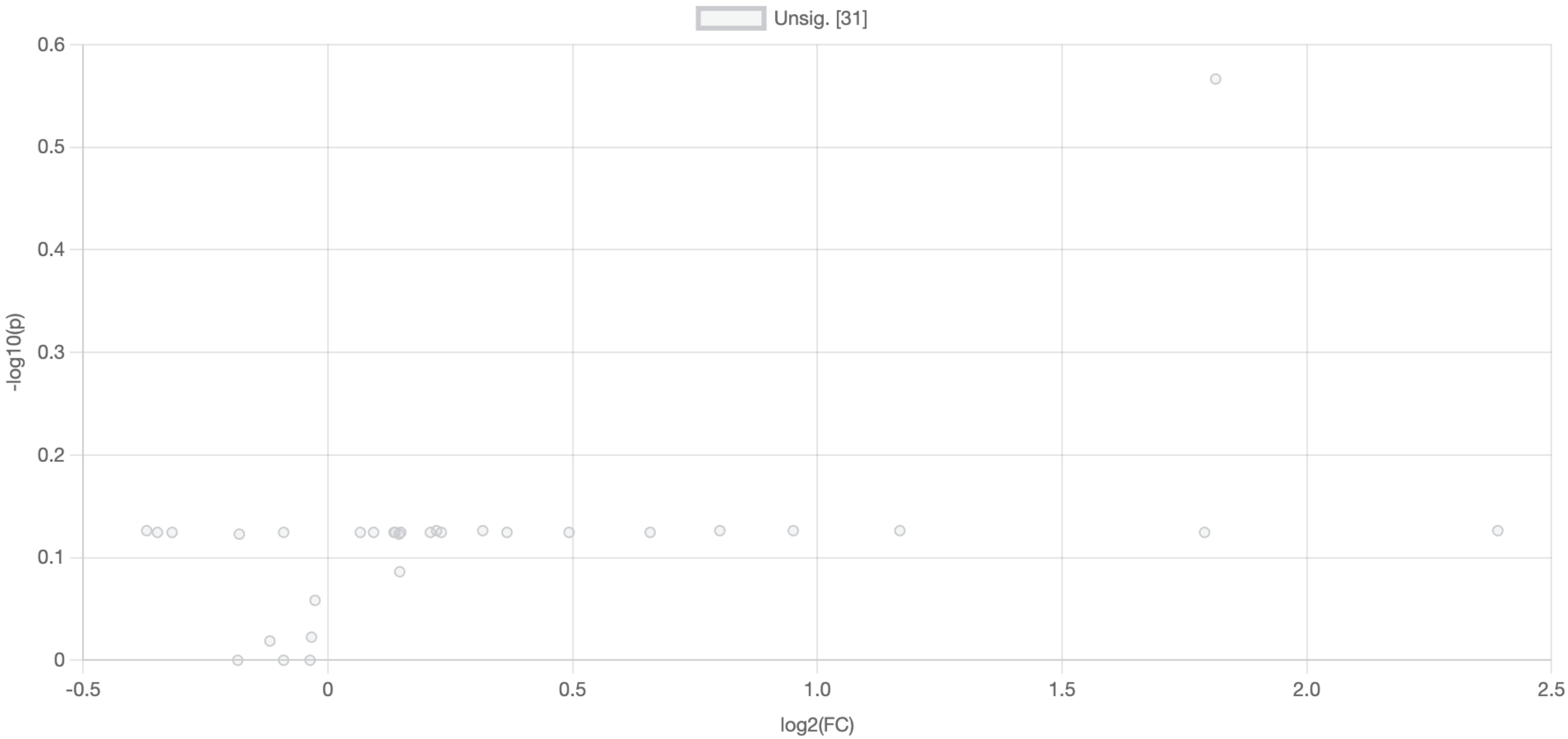


**Figure S8. Differential abundance analysis between COVID-19 with and without immunosuppressive therapy does not reveal significant differences in metabolite concentrations.** The analysis is based on the same metabolites as used in Figure 1. The ratio of mean concentration (“fold change (FC)”, Viral/Unknown) is plotted $log_2$ transformed on the x-axis, adjusted *p* value $log_{10}$ transformed (corrected for multple testing after Benjamini- Hochberg) on the y-axis. The threshold was set to FC>|1.5| and adjusted *p*-value <0.05.

| **Table S1.** Diagnostic criteria and additional clinical information | | | |
|---|---|---|---|
| **Diagnosis** | **Criteria** | **Disease activity** | **Medication at time of lumbar puncture[a]** |
| Acute COVID-19 (n=13) | Positive SARS-CoV-2 PCR or antigen test<br><br>Neurological deficit | Neurological deficits:<br>Polyneuropathy (n=5)<br>- Critical illness polyneuropathy (n=4)<br>- Guillain-Barré-Syndrome (n=1)<br>Altered mental status (n=8)<br>Seizures (n=3)<br>Focal neurological deficits (n=4)<br>- speech disturbances (n=2),<br>- visual disturbances (n=1),<br>- vertigo (n=3) | Anti-infective therapy (n=6)<br>- Ceftriaxone<br>- Azithromycin, voriconazole, piperacillin/tazobactam<br>- Meropenem, vancomycin<br>- Moxifloxacin<br>- Vancomycin, cefotaxime<br>- Acyclovir, caspofungin<br>Immunosuppressive therapy (n=4)<br>- Prednisolone, mycophenolate-mofetil, tacrolimus<br>- Dexamethasone<br>- Prednisolone, sirolimus, tacrolimus<br>- Dexamethasone<br>- Prednisolone<br>Antidepressants (n=2)<br>- Doxepin<br>- Sertraline |
| Post COVID-19 (n=3) | Past COVID-19 infection<br><br>Negative SARS-CoV-2 PCR or antigen test at time of lumbar puncture<br><br>Neurological deficits | Neurological deficits:<br>Post-Covid fatigue (n=1)<br>Amnesia (n=1),<br>Decreased muscle strength (n=1)<br>Paraplegia (n=1) | Immunosuppressive therapy (n=1)<br>- Prednisolone |
| Viral central CNS with known pathogen (n=10) | Detection of viral pathogen by PCR and/or ASI >1.5<br><br>Clinical meningitis[b]/ encephalitis[c]/ myelitis[d] | Meningitis 30%,<br>Encephalitis 20%,<br>Myelitis 10%,<br>Meningoencephalitis 40%. | Anti-infective therapy (n=2)<br>- Ceftriaxone, acyclovir, ampicillin/sulbactam<br>- Acyclovir, ceftriaxone, ampicillin<br>Anticonvulsive therapy (n=2)<br>- Levetiracetam<br>- Levetiracetam<br>Neuroleptic therapy (n=1)<br>- Melperon, olanzapine |

| | | | |
|---|---|---|---|
| Viral CNS infection without known pathogen (n=21) | Negative viral PCR and ASI>1.5<br><br>CSF changes typically for viral meningitis[b]/ encephalitis[c]/ myelitis[d]<br><br>Clinical meningitis/ encephalitis/ myelitis | Meningitis 43%, Encephalitis 38%, Myelitis 19% | Anti-infective therapy (n=1)<br>- Ceftriaxone, ampicillin, acyclovir<br>Immunosuppressive therapy (n=5)<br>- Prednisolone<br>- Prednisolone<br>- Cyclosporin A<br>- Prednisolone<br>- Dimethylfumarate<br>Antidepressants (n=2)<br>- Citalopram, mirtazapine<br>- Escitalopram<br>Anticonvulsive therapy (n=4)<br>- Valproic acid<br>- Lamotrigine<br>- Levetiracetam<br>- Valproic acid, levetiracetam |

[a] Excluding symptomatic therapy with analgesics and antipyretics, and medications for non-relevant conditions. [b] Putz K, Hayani K, Zar FA. Meningitis. Prim Care. 2013 Sep;40(3):707-26. [c] Ellul M, Solomon T. Acute encephalitis - diagnosis and management. Clin Med (Lond). 2018 Mar;18(2):155-159. [d] Irani DN. Aseptic meningitis and viral myelitis. Neurol Clin. 2008 Aug;26(3):635-55

| **Table S2.** Included metabolites | | |
|---|---|---|
| **Abbreviation** | **Name** | |
| Acylcarnitines (3) | | |
| C0 | Carnitine | |
| C4 | Butyrylcarnitine | |
| C5-OH (C3-DC-M) | Methylmalonylcarnitine | |
| | | |
| Amine Oxide (1) | | |
| TMAO | Trimethylamine N-oxide | |
| | | |
| Amino acid related (5) | | |
| Cit | Citrulline | |
| HCys | Homocysteine | |
| Orn | Ornitine | |
| SDMA | Symmetric dimethylarginine | |
| Taurine | Taurine | |
| | | |
| Amino acids (12) | | |
| Ala | Alanine | |
| Arg | Argenine | |
| Asn | Asparagine | |
| His | Histidine | |
| Ile | Isoleucine | |
| Leu | Leucine | |
| Lys | Lysine | |
| Met | Methionine | |
| Phe | Phenylalanine | |
| Ser | Serine | |
| Thr | Threonine | |
| Val | Valine | |
| | | |
| Biogenic amines (1) | | |
| Putrescine | Putrescine | |
| | | |
| Carboxylic acids (3) | | |
| AconAcid | Aconitic acid | |
| OH-GlutAcid | 3-Hydroxyglutaric acid | |
| Suc | Succinic acid | |
| | | |
| Nucleic base and related (2) | | |
| Hypoxanthine | Hypoxanthine | |
| Xanthine | Xanthine | |
| Ceramides (1) | | |

| | | |
|---|---|---|
| Cer(d18:1/18:0) | Cer(d18:1/18:0) | |
| | | |
| Hexosylceramides (2) | | |
| | Potential isomers | Lipid map ID |
| HexCer(d18:2/22:0) | GlcCer(d18:2/22:0) | LMSP0501AA37 |
| | GalCer(d18:2/22:0) | LMSP0501AC22 |
| Dihexosylceramides (1) | | |
| | Potential isomers | Lipid map ID |
| Hex2Cer(d18:1/18:0) | LacCer(d18:1/18:0) | LMSP0501AB04 |
| | Manβ1-4Glcβ-Cer(d18:1/18:0) | LMSP0501AD02 |
| | Galα1-4Galβ-Cer(d18:1/18:0) | LMSP0509AA02 |
| | | |
| Triglycerides (1) | | |
| | Potential isomers | Lipid map ID |
| TG(20:1_32:3) | TG(12:0/20:1(11Z)/20:3(8Z,11Z,14Z)) | LMGL03013595 |
| | TG(14:0/18:3(6Z,9Z,12Z)/20:1(11Z)) | LMGL03014406 |
| | TG(14:0/18:3(9Z,12Z,15Z)/20:1(11Z)) | LMGL03014423 |
| | TG(14:1(9Z)/18:2(9Z,12Z)/20:1(11Z)) | LMGL03014794 |
| | TG(15:1(9Z)/17:2(9Z,12Z)/20:1(11Z)) | LMGL03015463 |
| | TG(15:1(9Z)/17:2(9Z,12Z)/20:1(11Z)) | LMGL03015463 |
